# Acute respiratory distress syndrome driven by severe hypercalcemia and acute kidney injury: A case report and literature review of a rare, life-threatening complication 

**DOI:** 10.5414/CNCS110464

**Published:** 2022-01-24

**Authors:** Haresh Selvaskandan, Katherine Hull, Rachel Gregory, Daniel Pan, Thrasos Macriyiannis, Jenny Briggs, Catherine Richards, Catherine Mason, Jorge Jesus-Silva, Ricky Bell

**Affiliations:** University of Leicester, Leicester, UK

**Keywords:** hypercalcemia, parathyroid adenoma, acute respiratory distress syndrome, respiratory failure, acute kidney injury, parathyroidectomy, extra-corporeal membrane oxygenation support

## Abstract

Acute respiratory distress syndrome (ARDS) is a rare and under-reported complication of hypercalcemia, which often presents in conjunction with acute kidney injury (AKI). Unfamiliarity with the condition inevitably leads to management uncertainty, resulting in fatal outcomes. Early identification, however, confers a good prognosis. We report a case of a 40-year-old male who presented with severe hypercalcemia and AKI and rapidly deteriorated due to ARDS, with no evidence of cardiogenic pulmonary edema or fluid overload. Infection screens were negative. He died despite invasive ventilation and continuous venous-venous hemofiltration. His autopsy revealed extensive metastatic pulmonary calcifications and alveolar edema. We found only 10 other cases of hypercalcemia-induced ARDS in the literature, with only 2 patients surviving. We provide the first literature review on the subject to guide the management of this rare but fatal complication, which can be managed with good outcomes if considered early.

## Introduction 

**Figure 1:**
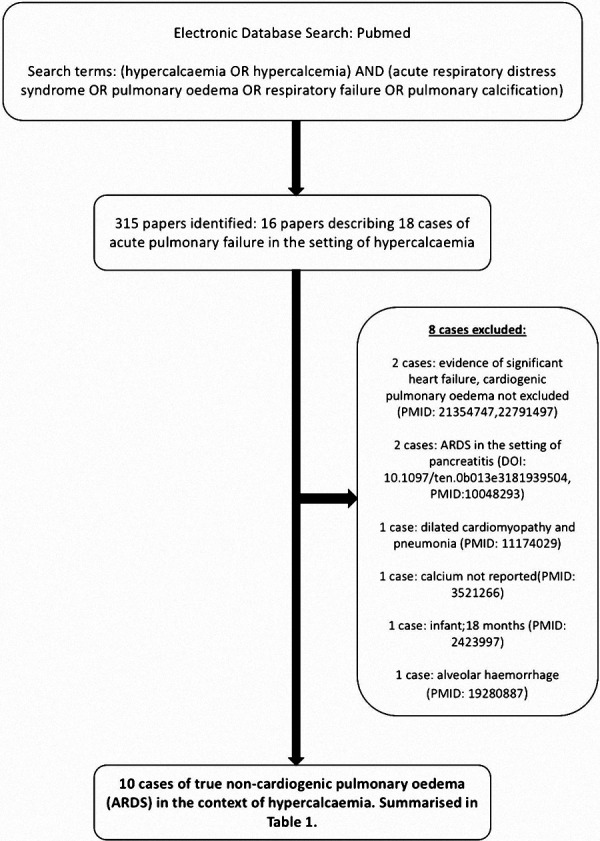
Summary of literature search performed.

Symptomatic hypercalcemia can manifest on a clinical spectrum, with acute respiratory distress syndrome (ARDS) being a particularly rare and likely under-reported presentation. Despite the prevalence of hypercalcemia, only 10 true cases of hypercalcemia-induced ARDS have been reported in the literature (Figure 1) (Table 1). This accounts for the relatively low level of awareness of the condition, and inevitably leads to diagnostic and management uncertainty in the context of a critically unwell patient, often leading to fatal outcomes (Table 1). However, cases identified and managed appropriately appear to have a good prognosis. We therefore find raising awareness of this differential in hypercalcemic patients with unexplained oxygen requirements, particularly in those with an acute kidney injury (AKI), to be an issue of great importance. We report a rare case of hypercalcemia-induced ARDS and offer the first literature review on the subject to guide management. 

## Case presentation 

A 40-year-old army reservist presented to the emergency department with a week of abdominal pain, constipation, bone pain, fatigue, and polyuria. He had a past medical history of epilepsy which was well controlled with sodium valproate. He had no significant family history of medical ailments. 

He was severely dehydrated on clinical examination. Despite this, he appeared comfortable and well, being apyrexial with a blood pressure of 156/101 mmHg, a heart rate of 90 beats per minute (bpm) and an oxygen saturation of 98% on room air. 

His initial investigations confirmed a severe hypercalcemia at 4.39 mmol/L (normal range 2.00 – 2.20), a phosphate of 1.85 mmol/L (1.12 – 1.45) and revealed a stage 3 AKI with a creatinine of 537 μmol/L (baseline 92 μmol/L 6 months prior). His white cell count and C-reactive protein were also raised at 15.9 × 10^9^/L (4.3 – 10) and 96 mg/L (< 5), respectively. A non-contrast CT scan of his renal tract excluded an obstructive hydronephrosis. His potassium and acid base balance were within normal limits. A parathyroid hormone measurement subsequently returned grossly elevated at > 200 pg/mL (10 – 65), confirming primary hyperparathyroidism as the driver of his hypercalcemia. 

His AKI and hypercalcemia were managed with aggressive intravenous fluids, and he was given broad spectrum antibiotic cover with intravenous co-amoxiclav, dose-adjusted for his renal function. His case was discussed with parathyroid surgeons, who agreed to list him for a parathyroidectomy following imaging and resolution of his AKI. Bisphosphonates were not commenced owing to his AKI, and calcimimetics were not commenced as he was deemed a candidate for a parathyroidectomy. 

**Figure 2:**
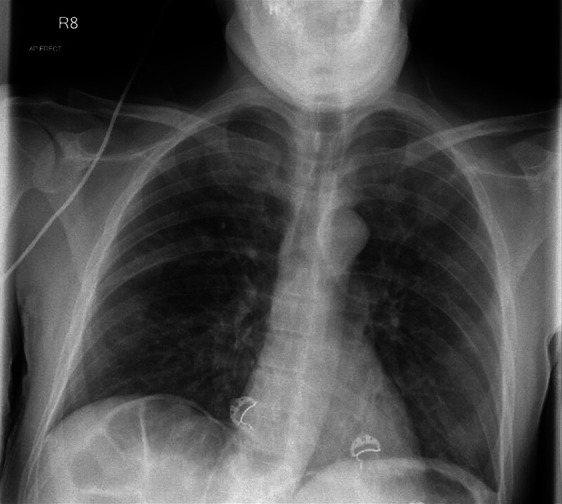
Chest radiograph performed at the time of initial oxygen requirement. Formally reported as showing minimal changes.

Approximately 24 hours into his admission, he desaturated to 92% on room air, managed with 4 L of supplemental oxygen. A chest radiograph performed at this time was reported as normal (Figure 2), prompting concerns of sub-clinical fluid overload. His intravenous fluids were stopped, and he was transferred to a high dependency unit for close observations. He continued to maintain a good urine output with minimal improvement in his AKI. 

His respiratory function rapidly deteriorated over the next 18 hours. He was transferred to the intensive care unit, where he was intubated and mechanically ventilated, following a failed trial of non-invasive bilevel positive airway pressure ventilation. 

At the time of intubation, he continued to maintain a good urine output and demonstrated several features in keeping with hypovolemia; echocardiography confirmed a collapsible inferior vena cava (IVC), with non-dilated, structurally and functionally normal ventricles, he was hypotensive (95/71 mmHg) and tachycardic (125 beats per minute), and had a narrow pulse pressure of 24 mmHg (normal range 40 – 60 mmHg). On examination there was no evidence of pitting edema and his jugular venous pressure (JVP) was depressed. Viral and atypical pneumonia screens as well as blood and urine cultures were all negative for infective causes. Three polymerase chain reaction (PCR) tests for SARs-CoV-2 were negative. 

**Figure 3:**
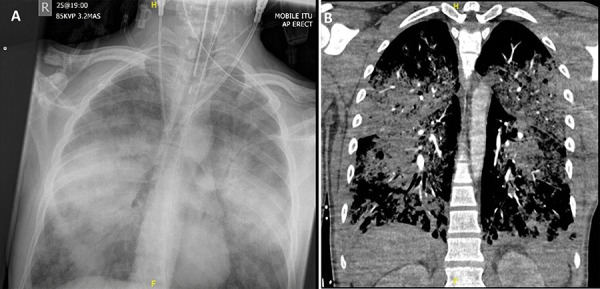
A: Repeat chest radiograph and B: CT pulmonary angiogram performed after intubation.

Imaging performed just after his intubation excluded a pulmonary embolus, but confirmed new bilateral pulmonary infiltrates which had developed over 18 hours (Figure 3). A “pepper-pot” skull and a large thyroid mass demonstrating no features of local invasion or metastases were also found on imaging. Repeat blood tests revealed a developing lactic acidosis, minor resolution of his hypercalcemia (calcium 3.93 mmol/L, phosphate 1.47 mmol/L), and little improvement in his AKI. These findings prompted broadening of his antibiotic therapy by converting to meropenem and clarithromycin, the initiation of dexamethasone to manage a presumed systemic inflammatory response syndrome, and continuous veno-venous hemofiltration was used to correct his calcium and acid-base abnormalities. Despite these efforts, he became progressively more hypoxic and died within a few hours of treatment escalation. 

**Figure 4:**
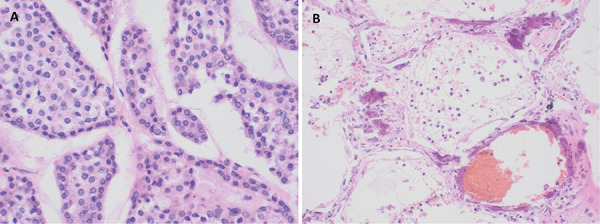
Post-mortem histopathology: A: parathyroid adenoma; B: metastatic pulmonary calcifications (H & E staining, × 400 magnification).

A post-mortem examination showed no evidence of vasculitis or infection in any organs and confirmed a structurally normal heart and a large parathyroid mass verified as a parathyroid adenoma on histology (Figure 4A). Alveolar edema was apparent on gross inspection of his lungs, with histological examination demonstrating metastatic pulmonary calcification (MPC) (Figure 4B). There was no pitting edema on examination. 

## Discussion 

We report an exceptionally rare case of hypercalcemia complicated by ARDS in the setting of an AKI. Upon review of the literature (10 cases identified over 50 years) (Figure 1) (Table 1), we believe this to be a treatable complication that is often misdiagnosed and undertreated owing to a lack of awareness. Here, we explore the evidence justifying this rare diagnosis in our case, and then review the literature to provide insights into possible mechanisms and best practice for management. 

Our patient experienced a rapid decline in pulmonary function over 18 hours, necessitating intubation and mechanical ventilation. Serial chest radiographs demonstrate the rapidity with which bilateral pulmonary infiltrates accumulated (Figures 2, Figure 3). There is sufficient evidence to confirm this was non-cardiogenic pulmonary edema; his heart was structurally normal on post mortem, and a bedside echocardiogram performed soon after intubation confirmed normal heart function. Despite aggressive fluid replacement, he maintained a good urine output and was in fact deemed to be fluid deplete on clinical examination upon intubation, making fluid overload an unlikely differential. This was suggested by a number of features; his IVC was collapsible, his JVP was depressed, he was hypotensive and tachycardic in the context of non-dilated ventricles and preserved systolic function, he had a narrow pulse pressure and continued to maintain a urine output, with no evidence of pitting edema. This was likely due to a sustained polyuria preceding his deterioration, caused by a loop diuretic-like effect and nephrogenic diabetes insipidus induced by severe hypercalcemia. This and the direct vasoconstrictive effects of calcium on glomerular capillaries were the likely drivers of his AKI [1]. 

His post-mortem found no infective changes in the lungs, and serology for parasites, fungi, and atypical bacterial infections were all negative. Furthermore, a respiratory viral screen performed from an endotracheal aspirate was negative, as were three PCR tests for the novel SARS-CoV-2 virus, each taken at least 1 day apart. There was no convincing evidence of pancreatitis in the context of his hypercalcemia. A sputum culture taken a day after his intubation grew a coliform species; this was likely a contaminant given he had blood cultures with no growth, and no evidence of a bacterial pneumonia on post-mortem. His post-mortem however did reveal MPC and alveolar edema, implicating hypercalcemia in the etiology of his ARDS. 

MPC refers to the inappropriate deposition of calcium in otherwise healthy lung tissue, commonly precipitating as whitlockite in contrast to hydroxyapatite usually found in bones, calcified blood vessels, and in calciphylaxis [2]. It is commonly related to an elevated calcium-phosphate product and can therefore be associated with a variety of conditions, including end-stage renal disease [3, 4]. A normal calcium-phosphate product is ~ 3.23 mmol^2^/L^2^, with values > 5.65 mmol^2^/L^2^ reported to predispose to MPC [4]. Our patient’s product was 9.1 mmol^2^/L^2^ on admission. The importance of an elevated calcium and phosphate in the genesis of MPC is highlighted by a number of key observations. First, primary hyperparathyroidism is an uncommon cause for MPC, postulated to be due to the renal phosphate wasting induced by elevated parathyroid hormone levels [2]. As in our case, MPC tends to develop in hyperparathyroidism only when phosphate excretion is impaired by renal dysfunction. Second, we note cases of ARDS developing in the context of hypercalcemia immediately after the administration of phosphate [3]. 

The lungs as a preferential site for calcium deposition has been proposed due to its relative alkalinity, induced by lower partial pressures of carbon dioxide locally. Calcium deposition is thought to begin in the basement membrane of alveolar cells, from where it extends to involve entire alveoli and their associated capillaries. This calcification can have variable clinical manifestations; the majority of cases appear to be only diagnosed on post-mortem examination for two reasons; 1) it is mostly asymptomatic and 2) it can only be detected with specialist imaging tools such as high-resolution CT or Tc99m-MDP scans, and is thus rarely identified incidentally [2]. In other cases, MPC can progressively and significantly impair gas exchange, accounting for why even intubation and mechanical ventilation is often insufficient to correct the hypoxemia that can develop [5, 6]. Of relevance to our case, MPC is known to develop rapidly in the setting of hypercalcemia and can predispose to non-cardiogenic pulmonary edema, as evidenced by our case and others (Table 1) [5, 6]. The mechanism by which ARDS occurs is unclear; a single study in rats demonstrated alveolar endothelial permeability was significantly increased in the context of hypercalcemia, mediated by inducible nitric oxide synthase, which was reversible with the correction of hypercalcemia with calcitonin [7]. 

We found a total of 18 cases of acute respiratory failure associated with hypercalcemia over the last 50 years, highlighting the rarity of the condition. As illustrated in Figure 1, we found only 10 cases where other causes of pulmonary edema were sufficiently excluded to allow for hypercalcemia-induced ARDS to be the most likely diagnosis. Of these, the only 2 patients to survive had acute parathyroidectomies, and were supported with renal replacement therapy and extra-corporeal membrane oxygenation (ECMO) therapy if needed [8, 9]. As discussed earlier, given the mechanism by which the calcifications envelope the alveoli, ECMO as a feasible therapeutic option can be physiologically justified. Indeed, 6 of the remaining 9 cases were intubated and mechanically ventilated but suffered persistent hypoxemia despite this, ultimately leading to death. We also note that in all reported cases including ours, the respiratory deterioration was rapid, occurring within 24 hours. 

Perhaps most crucially, if the underlying cause of the hypercalcemia can be treated, then both MPC and ARDS can rapidly resolve with correction of the calcium phosphate-product regardless of etiology with subsequent good outcomes [2, 9, 10, 11]. 

## Conclusion 

In conclusion, we report a presentation of hypercalcemic crisis and AKI complicated by rapid respiratory compromise. ARDS secondary to hypercalcemia was the likely cause. We propose a low index of suspicion for this complication in hypercalcemic patients with an AKI, who develop new and unexplained oxygen requirements. Should mechanical ventilation be warranted, we note a low success rate and advocate ECMO in the absence of contraindications. The calcium-phosphate product should be corrected. This can be achieved with calcimimetics, bisphosphonates, non-calcium phosphate binders, saline, calcitonin, and renal replacement therapy. This should be followed by treatment of the underlying etiology. Our literature review highlights a good prognosis in patients that are managed effectively in the acute setting. 

## Acknowledgment 

We would like to acknowledge our patient’s next of kin for consenting to having this case reported, and all healthcare professionals who were involved with the care of our patient. 

## Ethical approval 

As this was written as an anonymous case report, no ethical approval was required. 

## Consent for publication 

Consent was provided by our patient’s named next of kin. 

## Funding 

None to declare. 

## Conflict of interest 

None to declare. 

**Table 1.** Cases identified as true ARDS in the context of hypercalcemia. In all cases, hypercalcemia was originally managed with fluid replacement, and MPC was confirmed on histology if the patient did not survive.

No.	Age	Sex	Survived	Etiology of hypercalcemia	Comorbidities	Calcium mmol/L	Phosphate mmol/L	Calcium phosphate product mmol^2^/L^2^	Calcitonin	Bisphosphonates	Other management of hypercalcemia	Intubated	ECMO	Ref
1	46	F	Yes	Parathyroid adenoma	Schizophrenia	3.79	1.84	6.97	Yes	Yes	RRT	Yes	Yes	9
2	60	F	No	Parathyroid adenoma	CKD, HTN	3.69	1.32	4.87	No	No	Prednisolone, mithramycin, RRT	Yes	No	12
3	36	M	No	Parathyroid adenoma	–	5.44	1.55	8.43	No	No	Furosemide, phosphate, RRT	Yes	No	3
4^o^	66	F	No	Parathyroid adenoma	–	3.14	2.55	8.01	No	No	Furosemide, phosphate	Yes	No	3
5^o^	62	M	No	Diffuse B-cell lymphoma	–	3.64	1.16	4.22	Yes	No	Furosemide, phosphate, mithramycin	Yes	No	13
6	51	F	No	Myeloma	–	3.12	1.91	5.96	No	No	Plasma exchange, RRT	Yes	No	13
7	57	M	No	Myeloma	–	3.84	Not reported	–	Yes	No	Steroids	No	No	14
8*	41	F	No	Parathyroid adenoma	–	5.77	2.07	11.94	No	No	Phosphate	No	No	15
9	73	M	No	Parathyroid adenoma	–	5.19	Not reported	–	No	No	Furosemide, mithramycin	Yes	No	16
10	60	M	Yes	Parathyroid adenoma	HTN	> 5.09	Not reported	–	No	Yes	RRT	No	No	10
11	40	M	No	Parathyroid adenoma	Epilepsy, rheumatoid arthritis	4.38	2.07	9.06	Yes	No	Furosemide, steroids, RRT	Yes	No	Our case

*All cases included a chest radiograph report which demonstrated bilateral infiltrates of lung fields, except case 8, in which no chest radiograph was reported. ^o^Acute kidney injury was reported in all cases except cases 4 and 5. CKD = chronic kidney disease; ECMO = extra-corporeal mechanical oxygenation; HTN = hypertension; M = male; F = female; RRT = renal replacement therapy.
